# Supplementary material for: Stress relaxation rates of myocardium from failing and non-failing hearts
Source: Biomech Model Mechanobiol. 2024 Dec 31;24(1):265–80. doi: 10.1007/s10237-024-01909-4 (PMC11846740; doi:10.1007/s10237-024-01909-4)
Supplement: Supplementary file 1 — Supplementary file1 (DOCX 1746 KB) [file 10237_2024_1909_MOESM1_ESM.docx]

**Supplemental**


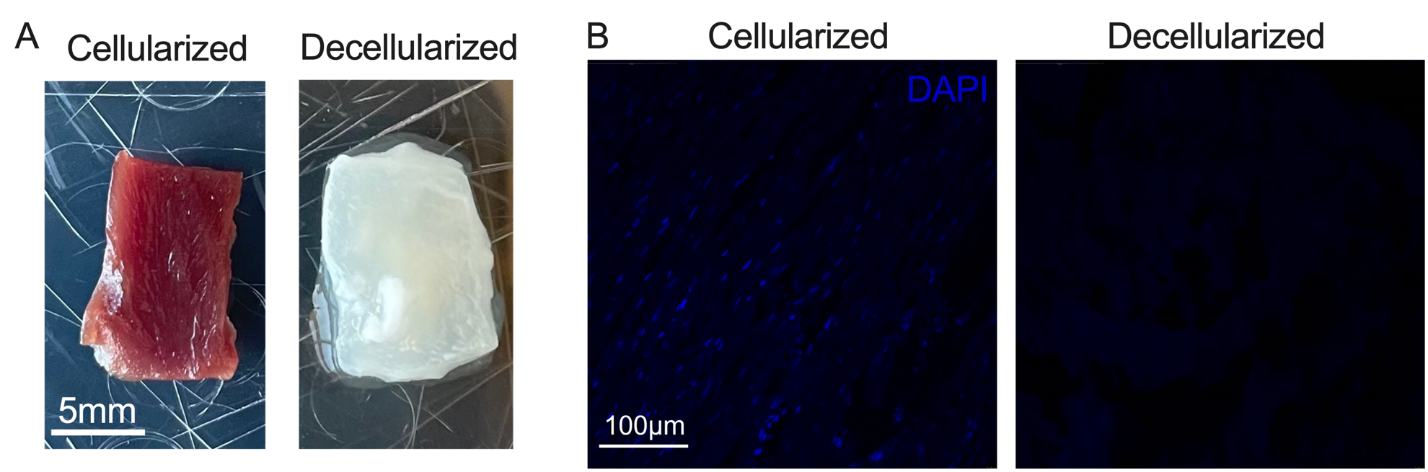


**Supplemental Figure 1. Decellularization was confirmed through a decrease in DAPI staining. (A)** Porcine tissue was decellularized, **(B-C)** verified by a depletion of DAPI staining. **(D)** 𝜏_1/2_ values of cellularized and decellularized porcine tissue.

**
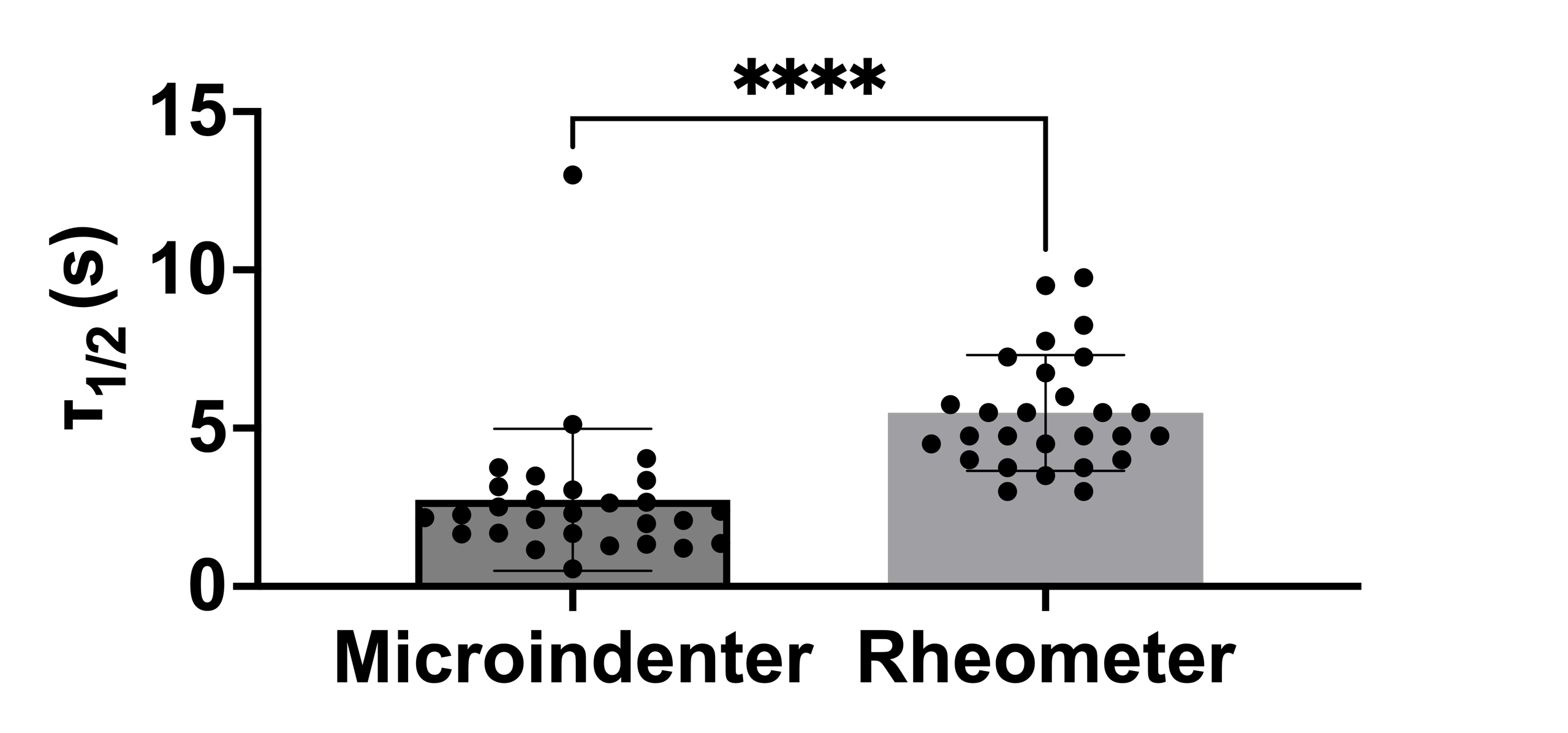
**

**Supplemental Figure 2. Porcine LV exhibited fast stress relaxation as measured on the rheometer and microindenter.** Measurement of 𝜏_1/2_ of porcine LV myocardium from the microindenter and rheometer in compression mode. Only statistics with p value less than or equal to 0.05 are shown. ****p ≤ 0.0001.


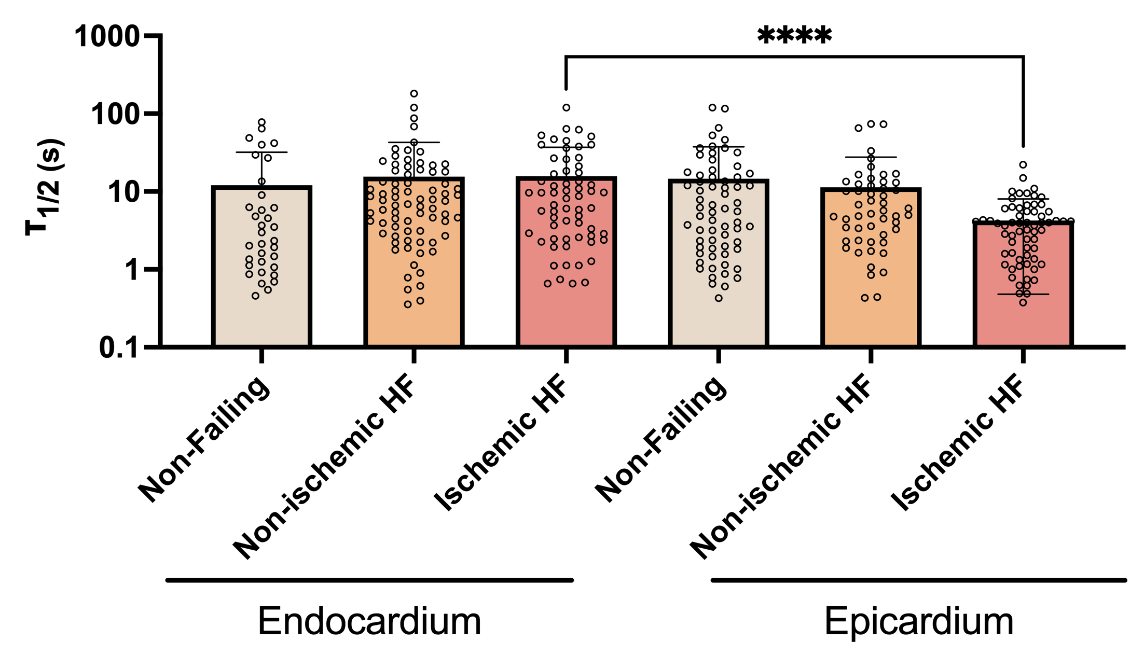


**Supplemental Figure 3. Ischemic HF group showed significant differences in 𝜏_1/2_ between endocardium and epicardium.** Statistical comparison between endocardium and epicardium. Only statistics with p value less than or equal to 0.05 are shown. ****p ≤ 0.0001.


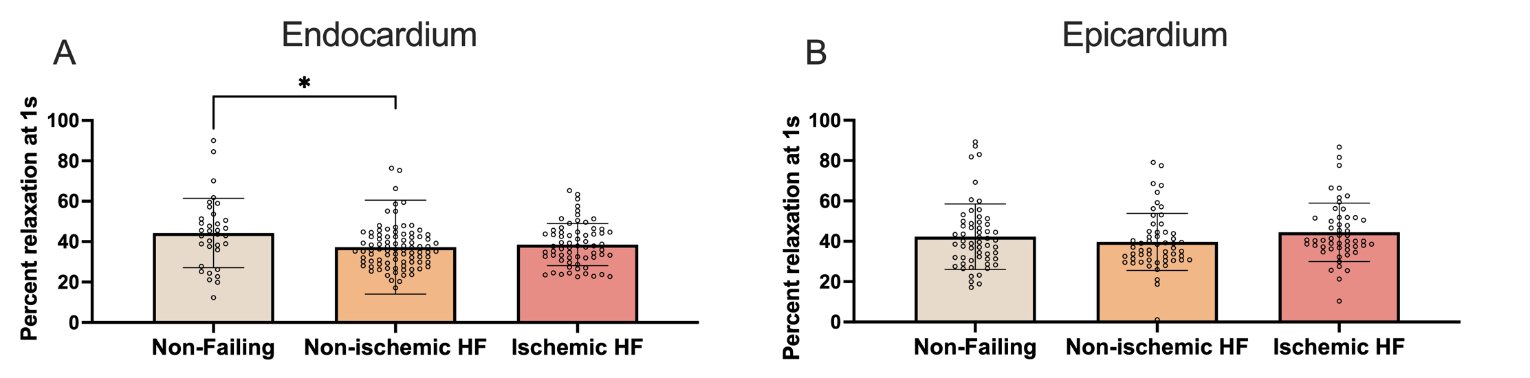


**Supplemental Figure 4. After 1s significant percent stress relaxation occurs in the epicardium and endocardium.** Percent stress relaxation occurring after 1s in the **(A)** endocardium and **(B)** epicardium. Only statistics with p value less than or equal to 0.05 are shown. *p ≤ 0.05.

**
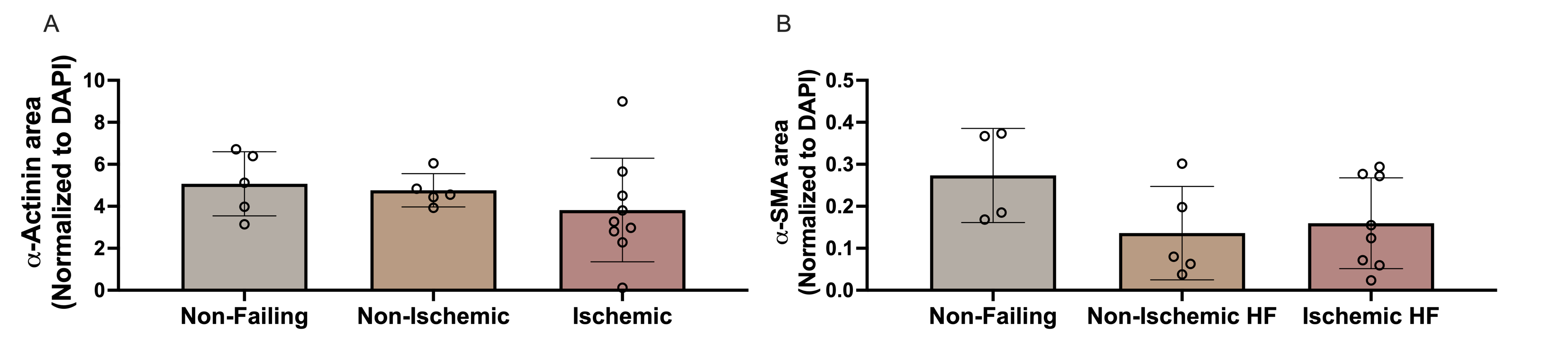
**

**Supplemental Figure 5. α-actinin and α-SMA staining quantification for the endocardium separated by clinical labels show no significance.** Quantification of the area of **(A)** α-actinin and **(B)** α-SMA normalized to DAPI for endocardium. Only statistics with p value less than or equal to 0.05 are shown.


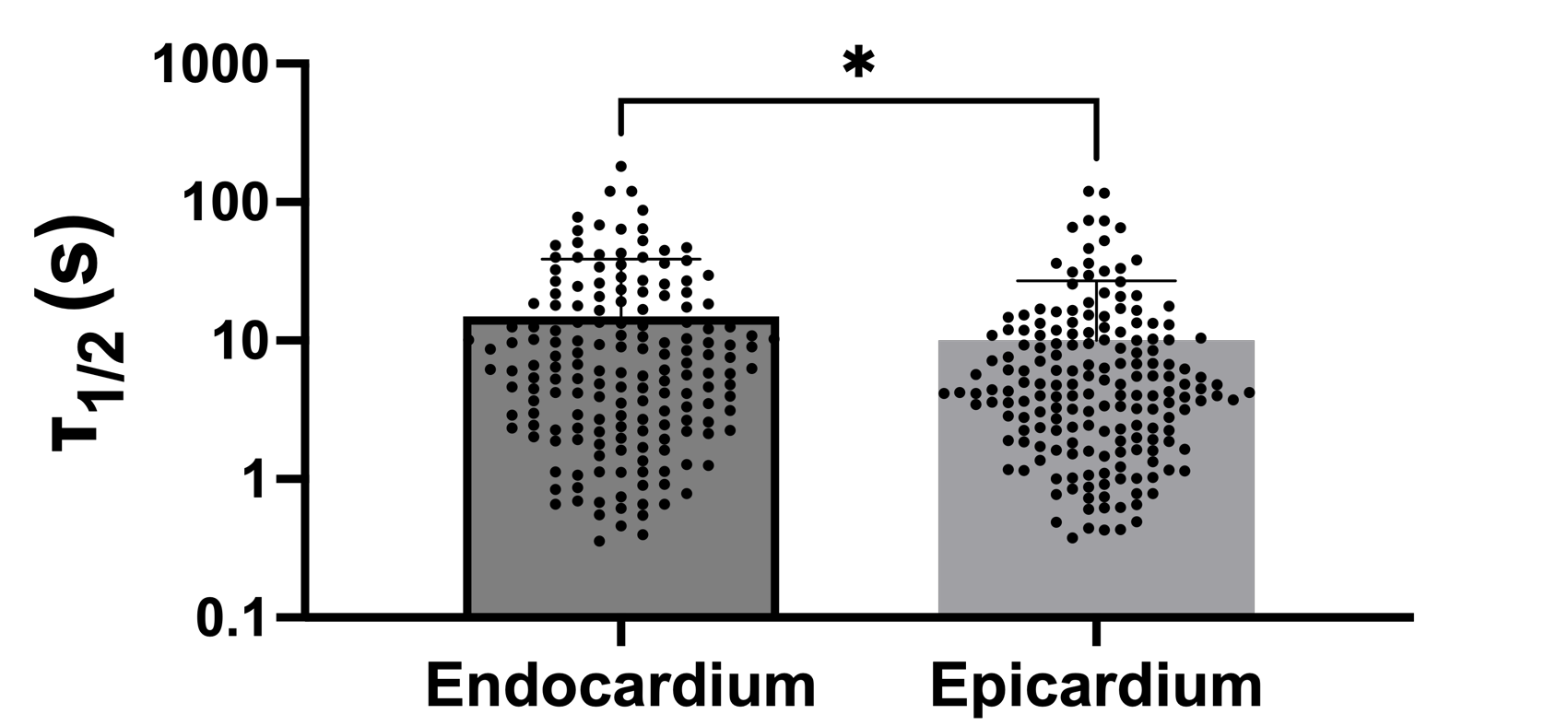


**Supplemental Figure 6. The endocardium shows significantly higher average 𝜏_1/2_ values compared to the epicardium.** Measurement of 𝜏_1/2_ between all human endocardium and epicardium samples. Only statistics with p value less than or equal to 0.05 are shown. *p ≤ 0.05.


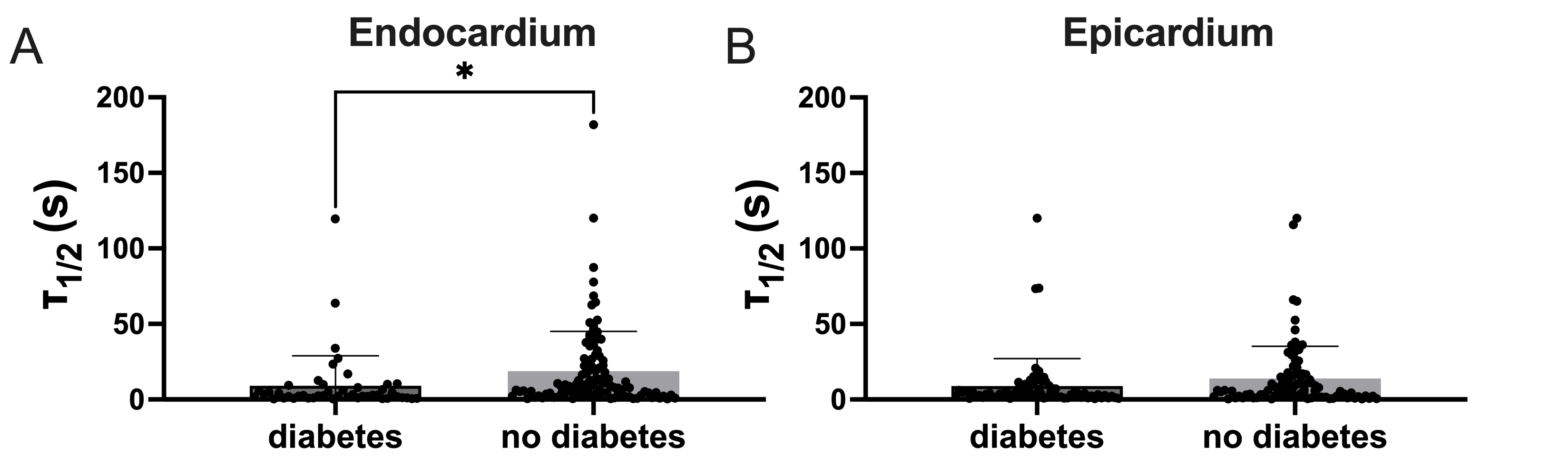


**Supplemental Figure 7. Patients with diabetes have lower 𝜏_1/2_ values than those without diabetes.** 𝜏_1/2_ values for **(A)** endocardium and **(B)** epicardium separated by patients who have or do not have diabetes. Only statistics with p value less than or equal to 0.05 are shown. *p ≤ 0.05.
